# Association of Second and Third Trimester Weight Gain in Pregnancy with Maternal and Fetal Outcomes

**DOI:** 10.1371/journal.pone.0054704

**Published:** 2013-01-30

**Authors:** Michele Drehmer, Bruce Bartholow Duncan, Gilberto Kac, Maria Inês Schmidt

**Affiliations:** 1 Graduate Studies Program in Epidemiology, Department of Social Medicine, Federal University of Rio Grande do Sul, Porto Alegre, Brazil; 2 Department of Social and Applied Nutrition, Rio de Janeiro Federal University, Josué de Castro Nutrition Institute, Rio de Janeiro, Brazil; Université de Montréal, Canada

## Abstract

**Objective:**

To investigate the association between weekly weight gain, during the second and third trimesters, classified according to the 2009 Institute of Medicine (IOM/NRC) recommendations, and maternal and fetal outcomes.

**Methods:**

Gestational weight gain was evaluated in 2,244 pregnant women of the Brazilian Study of Gestational Diabetes (Estudo Brasileiro do Diabetes Gestacional – EBDG). Outcomes were cesarean delivery, preterm birth and small or large for gestational age birth (SGA, LGA). Associations between inadequate weight gain and outcomes were estimated using robust Poisson regression adjusting for pre-pregnancy body mass index, trimester-specific weight gain, age, height, skin color, parity, education, smoking, alcohol consumption, gestational diabetes and hypertensive disorders in pregnancy.

**Results:**

In fully adjusted models, in the second trimester, insufficient weight gain was associated with SGA (relative risk [RR] 1.72, 95% confidence interval [CI] 1.26–2.33), and excessive weight gain with LGA (RR 1.64, 95% CI 1.16–2.31); in third trimester, excessive weight gain with preterm birth (RR 1.70, 95% CI 1.08–2.70) and cesarean delivery (RR 1.21, 95% CI 1.03–1.44). Women with less than recommended gestational weight gain in the 2^nd^ trimester had a lesser risk of cesarean deliveries (RR 0.82, 95% CI 0.71–0.96) than women with adequate gestational weight gain in this trimester.

**Conclusion:**

Though insufficient weight gain in the 3^rd^ trimester was not associated with adverse outcomes, other deviations from recommended weight gain during second and third trimester were associated with adverse pregnancy outcomes. These findings support, in part, the 2009 IOM/NRC recommendations for nutritional monitoring during pregnancy.

## Introduction

Nutritional interventions during prenatal care aimed at achieving adequate maternal weight gain have been shown to be effective [1]-[4]. However, debate remains concerning ideal gestational weight gain (GWG), and how to monitor it over the trimesters to reduce maternal and fetal complications. This debate has extended over at least seven decades. The difficulty in establishing recommendations is to strike a balance between a weight gain that is not so reduced as to cause low birth weight, restricted intrauterine growth and prematurity, yet which is not so high as to increase the chances of macrosomia, preeclampsia, cesarean section and gestational diabetes. The recommendations must be analyzed within the context of a world that is in the midst of an obesity epidemic, with a resultant higher percentage of women beginning their pregnancies while overweight, and major future risk of obesity for both the pregnant women and their offspring [5]-[10].

Studies have demonstrated a positive association between maternal second or third trimester weight gain and obstetric outcomes, for example, birth weight and gestational length [11], [12]. The pattern of GWG is related to maternal pre-pregnancy body mass index (BMI), mean weekly weight gain is generally higher in the second trimester. However, the pattern of GWG can vary depending on maternal ethnicity and age [13]. Surprisingly few studies have evaluated weight gain per trimester [13], [14].

In 2009, the Institute of Medicine (IOM/NRC) published new guidelines for gestational weight gain (total and per trimester) considering maternal and fetal outcomes during pregnancy and postpartum to determine adequate weight gain intervals, according to the pre-pregnancy BMI [13].

The new guideline differs from the one issued in 1990 in two ways. First, they are based on the World Health Organization (WHO) cutoff points for the BMI categories instead of those derived from Metropolitan Life Insurance tables. Second, the new guideline includes a specific, relatively narrow range of recommended gain for obese women [13]. Despite rapidly increasing obesity and proven weight retention after pregnancy, the IOM/NRC did not lower its recommended gains.

However, these new recommendations have not been sufficiently validated in different populations, especially the adequacy of recommendations of weekly weight gain in the 2^nd^ and 3^rd^ trimesters. Therefore, our paper aims to investigate adjusted associations of 2^nd^ and 3^rd^ trimester and overall weight gains, according to the cutoff points established by the IOM/NRC 2009, with the maternal and fetal outcomes mentioned above.

## Materials and Methods

The Brazilian Study of Gestational Diabetes (EBDG) is a multicenter cohort study with principal objective to evaluate American Diabetes Association (ADA) and World Health Organization (WHO) diagnostic criteria for gestational diabetes mellitus (GDM) against pregnancy outcomes [15]. It was conducted in health care units of the National Health System in six Brazilian cities: Porto Alegre, São Paulo, Rio de Janeiro, Salvador, Fortaleza and Manaus. The study methodology has been previously reported [15]. In brief, the study consecutively enrolled women 20 years or older from general prenatal clinics who were between 20 and 28 weeks of pregnancy and had no history of diabetes outside of pregnancy. The ethics committees of each institution involved approved the study. All participants provided their verbal informed consent after being informed about the studýs nature. All clinical investigations were conducted according to the principles expressed in the Declaration of Helsinki. This study occurred before the first regulatory guidelines for research involving humans in Brazil (National Council Resolution 196/96) that instituted mandatory written informed consent for all participants from the year 1996.

Our current investigation uses data from study phases I to III. Phase I consisted of standardized interviews and examinations, and glucose tolerance testing. The interview, performed at the prenatal clinicat enrollment, obtained information on maternal age, skin color, parity and education, as well as alcohol consumption and smoking. Weight and height were measured in duplicate according to standard protocol, and mean values were used in analyses [16]. Pre-pregnancy BMI was calculated using the reported pre-pregnancy weight and height measured at enrollment. Pre-pregnancy nutritional status was classified according to the current classification of the Institute of Medicine [13]. A 75 g oral glucose tolerance test was then performed between 24 and 30 weeks of pregnancy. Gestational diabetes was defined as blood glucose greater than or equal to 140 mg/dl two hours after intake, according to WHO criteria [17]. Hypertensive disorders were classified according to the National High Blood Pressure Education Program [18]. Data on clinical evolution, gestational weight gain and delivery were obtained through a review of medical records in study phases II and III. Phase II comprised all prenatal care, including maternal weight data from each prenatal consultation. Phase III involved data collection related to delivery, immediate postpartum period and the infants’ first hours of life.

**Figure 1:**
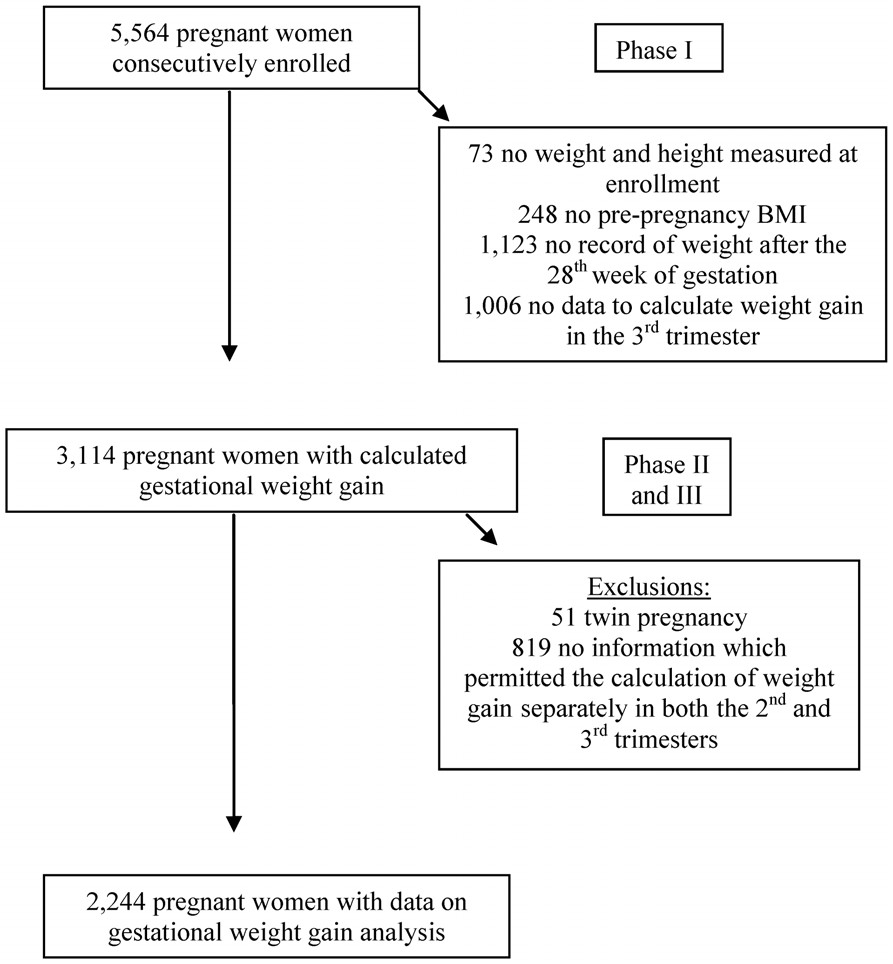
Study Flowchart.

From a total of 5,564 enrolled pregnant women, 73 did not have their weight and height measured at enrollment, 248 did not report pre-pregnancy weight, 1,123 had no clinic visit with recorded weight after the 28^th^ week of gestation and 1,006 had insufficient data to calculate weight gain in the third trimester, leaving 3,114 pregnant women with calculated gestational weight gain. We excluded an additional 51 participants due to multiple gestation and 819 due to not having information, which permitted the calculation of weight gain separately in both the second and third trimesters, resulting in a total of 2,244 for the weight gain analysis (Figure 1).

The trimesters were defined as first (less than 14 complete weeks), second (14–27 complete weeks) and third (28 complete weeks until delivery). The mean weekly weight gain in the second and third trimesters was estimated using the difference between the first and last weight record in the trimester divided by the number of weeks between the two observations, as previously reported [19].

Gestational weight gain was considered adequate in the 2^nd^ and 3^rd^ trimesters if the woman was within the range recommended by the 2009 IOM/NRC based on pre-pregnancy BMI: below 18.5 kg/m^2^, a gain between 0.44 and 0.58 kg/week; from 18.5 to 24.9 kg/m^2^, a gain from 0.35 to 0.50 kg/week; from 25 to 29.9 kg/m^2^, a gain from 0.23 to 0.33 kg/week; and greater than or equal to 30 kg/m^2^, a gain from 0.17 to 0.27 kg/week [3]. A total weight gain from 12.5 to 18 kg was considered adequate for women with pre-pregnancy BMI below 18.5 kg/m^2^; a gain from 11.5 to 16 kg for those with pre-pregnancy BMI from 18.5 to 24.9 kg/m^2^; a gain from 7 to 11.5 kg for those with pre-pregnancy BMI from 25 to 29.9 kg/m^2^; and a gain from 5 to 9 kg for those with pre-pregnancy BMI greater than or equal to 30 kg/m^2^ [13].

We estimated gestational age at delivery using an ultrasound exam performed before the 26^th^ week of gestation. When this information was not available, we used a hierarchical clinical criteria in the following order: any other ultrasound exam consistent with neonatal age estimation or reported last menstrual period; reported last menstrual period consistent with neonatal age estimation or uterine height; neonatal age estimation; ultrasound after week 26; uterine height; and finally, when no other criteria was available, last menstrual period.

Preterm birth outcome was considered as less than 37 weeks of gestation. Small for gestational age (SGA) was defined as birth weight below the 10^th^ percentile for gestational age in the EBDG study, considering those born alive with over 34 weeks of gestation and large for gestational age (LGA) as birth weight greater than the 90^th^ percentile in relation to gestational age.

Categorical characteristics of the sample are presented as absolute and relative frequencies. Pearson’s chi-square was used to test crude associations with weight gain per trimester, categorized as insufficient, adequate and excessive. Weight gain is expressed as a continuous variable with differences in weekly gains between the 2^nd^ and 3^rd^ trimesters being tested using the Wilcoxon matched-pairs signed ranks test.

To characterize the association of weight gain with each dichotomous obstetric outcome (cesarean section, preterm birth, SGA and LGA), Poisson regression models with robust variance were constructed with progressive inclusion of covariates. The covariates considered in the models were pre-pregnancy BMI, trimester-specific weight gain, age, height, skin color, parity, education, smoking, alcohol consumption, gestational diabetes and hypertensive disorders in pregnancy.

The criterion used to include these covariates was a p value <0.20 in the univariate analysis. Variables with p value <0.05 (Wald test) were maintained in the model, and those with higher p values were removed in decreasing order. Additionally, any potential confounder which changed the estimate of the relative risk for the association between weight gain and the outcome variable by >10% was kept in the model. Results were expressed as relative risk (RR) and 95% confidence interval (CI). We performed the data analyses using SPSS version 18 (SPSS Inc., Chicago, IL). The significance level was considered as 0.05.

## Results

Motherśmean (SD) age at enrollment was 27.9 (5.3) years and mean BMI 26.0 (3.9) kg/m^2^. Among the 2,244 women analyzed, 631 (28.1%) had insufficient and 975 (43.4%) excessive weight gain in the 2^nd^ trimester, and 874 (38.9%) had insufficient and 877 (39.1%) excessive weight gain during the 3^rd^ trimester. In relation to total weight gain during pregnancy, 750 (33.4%) women presented insufficient and 738 (32.9%) excessive weight gain. Gestational diabetes was diagnosed in 164 (7.7%; 95% CI 6.5–8.8).

As shown in Table 1, 48% of nulliparous women had excessive weight gain in the 2^nd^ trimester and 46% in the 3^rd^ trimester. Women with a higher level of education had a greater frequency of excessive weight gain in both the 2^nd^ (56%) and 3^rd^ trimesters (54%). A greater percentage of women with low pre-pregnancy weight had insufficient weight gains in the 2^nd^ (42%) and 3^rd^ (65%) trimesters while a greater percentage of women who were overweight (63%, 55% in the 2^nd^ and 3^rd^ trimesters, respectively) and obese (50%, 60% in the 2^nd^ and 3^rd^ trimesters, respectively) prior to conception had excessive weight gains.

**Table 1:** 2^nd^ and 3^rd^ trimester weight gain† according to maternal characteristics. Brazilian Study of Gestational Diabetes (EBDG) (n = 2,244).

Characteristic		2^nd^ trimester weight gain†			3^rd^ trimester weight gain†		
	n	Insufficient n (%)	Excessive n (%)	P value‡	Insufficient n (%)	Excessive n (%)	P value‡
**Age (years)**	2,244			0.612			0.055
<25	702	194 (27.6)	312 (44.4)		253 (36.0)	299 (42.6)	
25–29	722	195 (27.0)	322 (44.6)		269 (37.3)	290 (40.2)	
30–34	544	154 (28.3)	224 (41.2)		227 (41.7)	195 (35.8)	
≥35	276	88 (31.9)	117 (42.4)		125 (45.3)	93 (33.7)	
**Parity**	1,967			<0.001			<0.001
0	684	159 (23.2)	330 (48.2)		220 (32.2)	314 (45.9)	
1	632	180 (28.5)	272 (43.0)		243 (38.4)	245 (38.8)	
2	355	119 (33.5)	138 (38.9)		179 (50.4)	111 (31.3)	
≥3	296	108 (36.5)	108 (36.5)		149 (50.3)	83 (28.0)	
**Smoking habit** *	2,244			0.249			0.101
Never	1,321	375 (28.4)	569 (43.1)		505 (38.2)	518 (39.2)	
Past	546	144 (26.4)	257 (47.1)		200 (36.6)	228 (41.8)	
Current	377	112 (29.7)	149 (39.5)		169 (44.8)	131 (34.7)	
**Alcohol consumption** 2,211				0.829			0.174
Yes	774	221 (28.6)	339 (43.8)		289 (37.3)	325 (42.0)	
No	1437	403 (28.0)	619 (43.1)		571 (39.7)	545 (37.9)	
**Education (years)**	2235			<0.001			<0.001
<8	932	310 (33.3)	352 (37.8)		422 (45.3)	311 (33.4)	
8–11	1,081	279 (25.8)	494 (45.7)		393 (36.4)	444 (41.1)	
≥12	222	38 (17.1)	125 (56.3)		54 (24.3)	119 (53.6)	
**Pre-pregnancy BMI (kg/m^2^)**	2,244			<0.001			<0.001
<18.5	120	51 (42.5)	32 (26.7)		78 (65.0)	27 (22.5)	
18.5–24.9	1,479	430 (29.1)	553 (37.4)		614 (41.5)	487 (32.9)	
25.0–29.9	505	101 (20.0)	320 (63.4)		151 (29.9)	279 (55.2)	
≥30.0	140	49 (35.0)	70 (50.0)		31 (22.1)	84 (60.0)	

† as defined by Institute of Medicine 2009 recommendations (IOM/NRC 2009).

‡ P value refers to chi-square test for proportions.

* Smoking habit: Never smoking, quitting smoking prior to pregnancy, continued to smoke during pregnancy.

BMI: Body mass index (kg/m^2^).

Table 2 shows that mean weekly weight gain was higher in the 2^nd^ than in the 3^rd^ trimester (except for obese women). The means were greater than or equal to the maximum limit recommended by the IOM/NRC per week in the 2^nd^ and 3^rd^ trimesters among obese and overweight women. Pregnant women with low pre-pregnancy weight had a mean weight gain in the 2^nd^ trimester near the lower limit recommended, and below this limit in the 3^rd^ trimester.

**Table 2:** 2^nd^ and 3^rd^ trimester gestational weekly weight gain according to pre-pregnancy body mass index category according Institute of Medicine/NRC 2009. Brazilian Study of Gestational Diabetes (EBDG) (n = 2,244).

	Weekly weight gain (kg/week)			
	IOM/NRC 2009 Recommendation	2^nd^ trimester	3^rd^ trimester	P value†
**Pre-pregnancy BMI (kg/m^2^)**		**Mean (SD)**		
<18.5 (n = 120)	0.44 - 0.58	0.46 (0.28)	0.37 (0.32)	0.002
18.5–24.9 (n = 1479)	0.35 - 0.50	0.45 (0.25)	0.41 (0.29)	<0.001
25.0–29.9 (n = 505)	0.23 - 0.33	0.43 (0.28)	0.39 (0.31)	0.013
≥30.0 (n = 140)	0.17 - 0.27	0.27 (0.27)	0.37 (0.28)	<0.001

SD, standard deviation.

BMI, body mass index.

† Wilcoxon signed rank sum test for difference between 2^nd^ and 3^rd^ trimester.

Cesarean section occurred in 839 (37.8%, 95% CI 35.8–39.8%) of the pregnancies, preterm birth in 170 (7.6%, 6.5–8.7%), SGA birth in 208 (9.5%, 8.3–10.7%) and LGA birth in 230 (10.5%, 9.2–11.8%).

**Figure 2:**
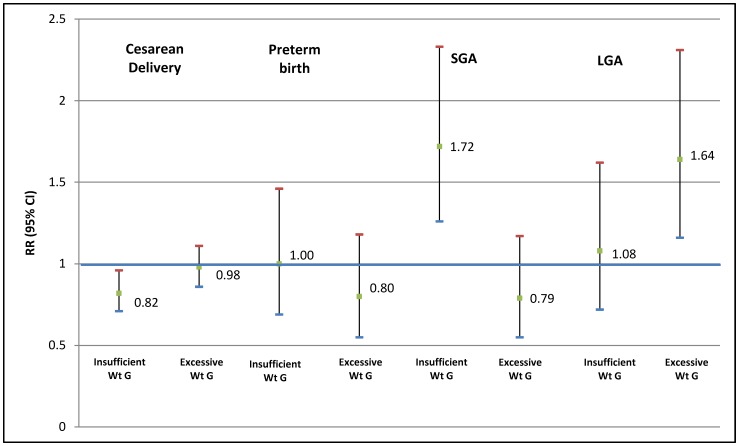
Adjusted* relative risks of cesarean delivery, preterm birth, small for gestational age (SGA) and large for gestational age (LGA) birth for women below and above the Institute of Medicine (IOM/NRC 2009) guidelines for 2^nd^ trimester weekly gestational weight gain. Brazilian Study of Gestational Diabetes (EBDG) (n = 2,244). *adjusted through Poisson regression with a robust error variance for education, age, skin color, parity, hypertensive disorders, diabetes, height, smoking, alcohol consumption and 3^rd^ trimester weight gain. Wt G: weight gain.

**Figure 3:**
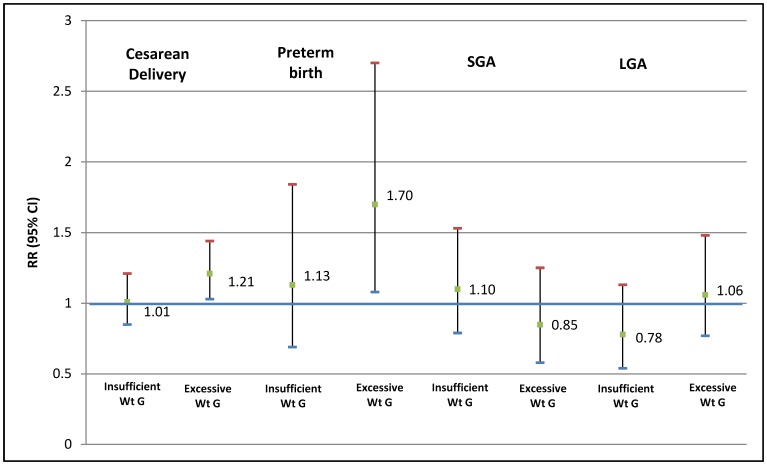
Adjusted* relative risks of cesarean delivery, preterm birth, small for gestational age (SGA) and large for gestational age (LGA) birth for women below and above the Institute of Medicine (IOM/NRC 2009) guidelines for 3^rd^ trimester weekly gestational weight gain. Brazilian Study of Gestational Diabetes (EBDG) (n = 2,244). *adjusted through Poisson regression with a robust error variance for education, age, skin color, parity, hypertensive disorders, diabetes, height, smoking, alcohol consumption and 2^nd^ trimester weight gain. Wt G: weight gain.

Insufficient total weight gain was associated with a lower risk of cesarean section (RR 0.78, 95% CI 0.68–0.91) and a higher risk of preterm birth (RR 1.45, 95% CI 1.00–2.11) and SGA (RR 1.60, 95% CI 1.19–2.15). In contrast, excessive total weight gain was associated with higher risk of cesarean section (RR 1.17, 95% CI 1.04–1.33) and LGA (RR 2.12, 95% CI 1.55–2.89) and with lower risk of SGA (RR 0.53, 95% CI 0.35–0.81) (Table 3 and Table 4). For women with insufficient weight gain in the 2^nd^ trimester, a higher risk of SGA (RR 1.72, 1.26–2.33) and a lower risk of cesarean section (RR 0.82, 95% CI 0.71–0.96) were observed. No association was found with insufficient weight gain in the final trimester. For women with excessive weight gain in the second trimester, we found a greater risk of LGA birth (RR 1.64, 95% CI 1.16–2.31), and a greater risk of pre-term birth (RR 1.70, 95% CI 1.08–2.70) and cesarean section (RR 1.21, 95% CI 1.03–1.44) when it occurred in the third trimester (Figure 2 and Figure 3).

**Table 3:** Association of cesarean section and preterm birth with weekly gestational weight gain in the 2^nd^ and 3^rd^ trimesters and total weight gain (according to Institute of Medicine, 2009, categories). Brazilian Study of Gestational Diabetes (EBDG).

	Cesarean section (n = 2,219)†		Preterm birth (n = 2,241)†	
Weekly gestational weight gain	Model 1Crude RR (95% CI)	Model 2*Adjusted RR (95% CI)	Model 1Crude RR (95% CI)	Model 2**Adjusted RR (95% CI)
**2^nd^ trimester**				
Insufficient	0.81 (0.69–0.94^)^ ‡	0.82 (0.71–0.96)‡	1.28 (0.89–1.83)	1.00 (0.69–1.46)
Excessive	1.05 (0.93–1.19)	0.98 (0.86–1.11)	0.79 (0.54–1.13)	0.80 (0.55–1.18)
**3^rd^ trimester**				
Insufficient	0.90 (0.77–1.04)	1.01 (0.85–1.21)	1.40 (0.91–2.16)	1.13 (0.69–1.84)
Excessive	1.19 (1.03–1.36)‡	1.21 (1.03–1.44)‡	1.58 (1.04–2.42)‡	1.70 (1.08–2.70)‡
**Total weight gain**				
Insufficient	0.74 (0.64–0.86)‡	0.78 (0.68–0.91)‡	1.42 (1.01–2.01)‡	1.45 (1.00–2.11)‡
Excessive	1.26 (1.12–1.42)‡	1.17 (1.04–1.33)‡	0.95 (0.64–1.38)	1.18 (0.74–1.67)

† Differences from 2,244 due to lack of data on the specific outcomes.

‡ Significant relative risk.

* Cesarean section: Model 2 (2^nd^ trimester) = Model 1+ pre-pregnancy BMI, age, education, parity, hypertensive disorders, height and birth weight. Skin color and 3^rd^ trimester weekly weight gain were removed from the model 2 due to lack of statistical significance. Model 2 (3^rd^ trimester) = Model 1+ pre-pregnancy BMI, age, education, parity, hypertensive disorders, diabetes, height, birth weight, alcohol consumption, gestational age at delivery and 2^nd^ trimester gestational weight gain. Skin color and smoking were removed from the model 2 due to lack of statistical significance. Model 2 (total weight gain) = Model 1+ pre-pregnancy BMI, age, education, parity, hypertensive disorders height and birth weight. Skin color, alcohol consumption, diabetes, smoking and gestational age at delivery were removed from the model 2 due to lack of statistical significance.

** Preterm birth: Model 2 (2^nd^ trimester) = Model 1+ pre-pregnancy BMI, education, hypertensive disorders, diabetes, 3^rd^ trimester gestational weight gain and birth weight. Age, height and parity were removed from the model 2 due to lack of statistical significance. Model 2 (3^rd^ trimester) = Model 1+ pre-pregnancy BMI, education, diabetes, birth weight and 2^nd^trimester gestational weight gain. Hypertensive disorders was removed due to lack of statistical significance.

Model 2 (total weight gain) = Model 1+ pre-pregnancy BMI, diabetes, hypertensive disorders, birth weight. Education was removed due to lack of statistical significance.

RR = risk relative; Reference category = Adequate weight gain.

**Table 4:** Association of small for gestational age and large for gestational age birth with weekly gestational weight gain in 2^nd^ and 3^rd^ trimesters and with total weight gain (according to Institute of Medicine, 2009 categories).

	Small for gestational age (n = 2,191)†			Large for gestational age (n = 2,191)†	
Weekly gestationalweight gain	Model 1Crude RR (95% CI)	Model 2*Adjusted RR (95% CI)	Model 1Crude RR (CI 95%)		Model 2**Adjusted RR (95% CI)
**2^nd^ trimester**					
Insufficient	1.71 (1.25–2.34)‡	1.72 (1.26–2.33)‡	1.15 (0.78–1.69)		1.08 (0.72–1.62)
Excessive	0.66 (0.46–0.94)‡	0.79 (0.55–1.17)	2.00 (1.45–2.77)‡		1.64 (1.16–2.31)‡
**3^rd^ trimester**					
Insufficient	1.30 (0.93–1.81)	1.10 (0.79–1.53)	0.74 (0.52–1.05)		0.78 (0.54–1.13)
Excessive	0.73 (0.50–1.06)	0.85 (0.58–1.25)	1.27 (0.93–1.74)		1.06 (0.77–1.48)
**Total weight gain**					
Insufficient	1.41 (1.06–1.87)‡	1.60 (1.19–2.15)‡	0.99 (0.69–1.43)		0.92 (0.63–1.34)
Excessive	0.51 (0.34–0.75)‡	0.53 (0.35–0.81)‡	2.24 (1.65–3.03)‡		2.12 (1.55–2.89)‡

† Differences from 2,244 due to lack of data on specific outcome (small and large for gestational age).

‡ Significant relative risk.

* Small-for-gestational-age: Model 2 (2^nd^ trimester) = Model 1+ pre-pregnancy BMI, hypertensive disorders, height, smoking and 3^rd^ trimester weight gain. Age, skin color, parity and diabetes were removed due to lack of statistical significance. Model 2 (3^rd^ trimester) = Model 1+ pre-pregnancy BMI, hypertensive disorders, height, smoking and 2^nd^ trimester weight gain. Age, skin color, parity and diabetes were removed due to lack of statistical significance. Model 2 (total weight gain) = Model 1+ pre-pregnancy BMI, hypertensive disorders, smoking and diabetes. Age, skin color, parity and height were removed due to lack of statistical significance.

** Large-for-gestational-age: Model 2(2^nd^ trimester) = Model 1+ pre-pregnancy BMI, 3^rd^ trimester weight gain, diabetes, smoking and height. Age, skin color, parity and education were removed due to lack of statistical significance. Model 2 (3^rd^ trimester) = Model 1+ pre-pregnancy BMI, height, 2^nd^ trimester weight gain, smoking and diabetes. Age, parity and education were removed due to lack of statistical significance. Model 2 (total weight gain) = Model 1+ pre-pregnancy BMI, smoking, diabetes and height. Age, parity, and education were removed due to lack of statistical significance.

Reference category =  Adequate weight gain.

Brazilian Study of Gestational Diabetes (EBDG).

## Discussion

The EBDG, a cohort study designed and carried out in the 1990s, remains the largest study of the association of gestational weight gain with maternal and infant obstetric outcomes in Brazilian women. The present analysis shows that gestational weight gain outside of the range recommended in the 2009 IOM/NRC guidelines, impacts differently on maternal and fetal outcomes, depending on the pregnancy trimester of the gain. Weight gain in the 2^nd^ and 3^rd^ trimester and total weight gain showed associations with birth weight, preterm birth and cesarean section, independent of pre-pregnancy BMI and maternal characteristics. Extremes of infant birth weight were more associated with weight gain in the 2^nd^ trimester, whereas risk of preterm birth and cesarean section with excessive weight gain in the 3^rd^ trimester. These findings support that monitoring the weight during pregnancy may be one of the keys to avoid adverse fetal and maternal outcomes, in so far as weight deviations can be identified and corrected in the course of pregnancy [10], but according to our data, insufficient weight gain in the third trimester produced no harm in obstetrical outcomes (birth weight, preterm birth and cesarean section).

The mean gestational weight gain in the 2^nd^ trimester was higher than in the 3^rd^, except for women with pre-pregnancy obesity. This is in agreement with studies that show greater means of weekly gain when compared to the 1^st^ and 3^rd^ trimester and higher correlations with birthweight [12], [13], [20]. Hickey and colleagues (1996) demonstrated that low gestational weight gain, particularly in the 2^nd^ trimester, reduces significantly the weight of the infant [11]. Fetal growth in the 2^nd^ trimester is indeed faster compared to the other trimesters, and more subject to interferences related to maternal nutrition. Low weight gain during this period doubled the risk of restricted intrauterine growth [21].

The main paradox of the relationship between gestational weight gain and birth weight is the playoff of benefits of greater maternal gain in terms of reducing SGA births and harm in terms of increasing LGA births. The magnitude of the protective association of weight gain above the IOM/NRC in relation to the SGA outcome is supported by strong scientific evidence, reaching values of up to 52% in SGA reduction among women with excessive gestational weight gain (95% CI 0.45–0.50) [22]. The present study demonstrated that excessive total weight gain is associated with a 42% (CI 0.38–0.87) reduction in SGA. Conversely, it is known that the maternal consequences of excessive gestational weight gain are unfavorable since it increases the risk of gestational diabetes, hypertensive disorders, complications of delivery and postpartum weight retention [8] **,** as well as leads to birth of LGA infants [23]-[27].

In the present study, women who had excessive weight gain in the 2^nd^ trimester regardless of pre-pregnancy BMI, 3^rd^ trimester weight gain, height, diabetes and presence of smoking habit, had higher risk of LGA. It is known that infants with very large birth weight for their gestational age have twice the risk of neonatal mortality and are more prone to develop obesity [26].

Gestational weight gain appears to be inadequately monitored in primary care services. In a more recent Brazilian cohort study investigating 667 women, the incidence of excessive or insufficient weight gain was high (45% and 26%, respectively) [28]. Among women in that study with pre-pregnancy obesity, the vast majority (81%) showed inappropriate weight gain, either insufficient (24%) or excessive (57%). Two apparently paradoxical findings were present. First, having few prenatal visits was a risk factor for insufficient weight gain, but was a protective factor against excessive weight gain. Secondly, starting pregnancy when overweight or obese proved to be a risk factor for excessive weight gain, while starting underweight was not a risk factor for insufficient weight gain during pregnancy.

Another consequence of high weight gain is a greater chance of cesarean delivery [22], [29], [30]. In Brazil, almost half of all births occurred this way (47%). The cesarean rate in the public healthcare system is 35% and in the private system reaches 80% [31]. It has been shown in previous work that excessive weight gain during pregnancy increased in 40% the chance of cesarean delivery, after adjusting for infant birth weight (95% CI 1.22–1.59) [32]. This previous study highlights that despite macrosomia being a strong predictor of cesarean section, excessive weight gain was an independent risk factor for this outcome, and it also argues that from the 288,000 cesarean deliveries performed in the U.S. every year, approximately 64,000 could be prevented if women had weight gain according to the IOM/NRC recommendations [32]. The present study is in accordance with these findings regarding total and especially 3^rd^ trimester excessive weight gain. Our finding that insufficient gestational weight gain protects against cesarean section is in contrast with that reported previously [10]. This issue has not been broadly discussed in the literature [6],[22].

An intriguing finding in this study was that excessive weight gain in 3^rd^ trimester was a risk factor for preterm birth. There are no clear biologic mechanisms for the link between excessive pregnancy weight gain and preterm delivery. A possible explanation might be that this excessive weight gain may be simply a marker for edema, in turn a marker for preeclampsia [33]-[35], which is associated with preterm delivery. However, in the present study, this association was adjusted for hypertensive disorders and others confounding variables. Only one study found the association restricted to excessive third-trimester weight gain, though only among women with normal pre-pregnancy BMI [35].

The present study has some limitations. Pre-pregnancy weight was reported by the woman. However, this way of obtaining this information is widely used in literature [13], [19], [20]. The correlation between the reported and the measured weight is very strong, reaching values up to 0.97 in the population of Porto Alegre, being a little lower at the extremes of the nutritional status [36]. Information about 2^nd^, 3^rd^ trimester and total weight gain was only possible to be performed in 40.3% of the initial sample due to insufficient data in the medical record files of the prenatal clinics and hospitals. Though many women ended up being excluded due to lack of data, those excluded had similar maternal characteristics at enrollment. Another limitation is that the weights used to calculate weight gain were retrieved from medical records. Data collection of medical records may lead to results which are not significant in associations of maternal nutritional state and gestational weight gain with obstetric outcomes [14]. Weight at enrollment was the only weight obtained in duplicate. Lastly, residual confounding by socioeconomic status may have occurred, since mother’s education and skin color were the only variables representing socioeconomic status in the analysis. In the Brazilian context, in general, blacks have greater social vulnerability than whites, permitting skin color to be used as a proxy for socio-economic status [37].

Given the limited availability of standardized prospective cohort studies in pregnancy, the data presented here contribute to the knowledge of this area, especially in the Brazilian context. In conclusion, both insufficient and excessive weight gain, evaluated according to the North American guidelines released by the IOM/NRC in 2009, are indicators of risk for adverse obstetric outcomes for both mother and infant. However the use of these North American recommendations to promote adequate pregnancy outcomes for Brazilian women is not supported for pregnant women with respect to insufficient weight gain in the 3^rd^ trimester as insufficient gain did not result in any adverse maternal and fetal outcome. If further studies confirm this lack of association between the low weight gain in the last trimester and obstetric events, independent of pre-pregnancy BMI, consideration should be given to a lower recommended weight gain in this trimester.
